# How model guided photosynthetic bioengineering can help to feed the world

**DOI:** 10.1093/plphys/kiad563

**Published:** 2023-11-01

**Authors:** Henning Kirst

**Affiliations:** Assistant Features Editor, Plant Physiology, American Society of Plant Biologists; Departamento de Genética, Campus de Excelencia Internacional Agroalimentario ceiA3, Universidad de Córdoba, 14071 Córdoba, Spain; Instituto Maimónides de Investigación Biomédica de Córdoba (IMIBIC), 14004 Córdoba, Spain

The Green Revolution has substantially increased crop yields. However, agricultural productivity is plateauing (Zhu et al. 2010). Given the increasing demand from world population growth (van Dijk et al. 2021) and the negative impact of climate change on agricultural productivity (Gornall et al. 2010; van Dijk et al. 2021), new technologies are needed to meet these challenges. A promising approach is to increase photosynthetic productivity that has not been targeted directly in the Green Revolution. Using bioengineered components of the photosynthetic machinery, significant yield increases can be achieved to further improve agricultural productivity (Ort et al. 2015; Leister 2023). For example, photosynthetic organisms use the energy of sunlight inefficiently at high light intensities. Only about 30% of bright sunlight is used for photosynthesis (Melis 2009). This can be significantly improved by limiting the light absorption to only the amount that they can use for photosynthesis. The excess light is passing through, enabling more cells to photosynthesize. In fact, this counterintuitive approach has increased photosynthetic productivity in a wide variety of organisms (Kirst et al. 2014, 2017; Friedland et al. 2019; Slattery and Ort 2021).

Another approach is to accelerate the plants' capacity to react to fluctuating light conditions, which can be caused by clouds for example. Tobacco plants bioengineered to faster relax photoprotection and thereby acclimate faster to changing light conditions than wild type plants accumulated 15% more biomass in a growth season (Kromdijk et al. 2016). However, the same modifications engineered into *Arabidopsis thaliana* had the exact opposite effect (Garcia-Molina and Leister 2020). Because photosynthesis is a sequence of reactions, its rate is limited by the slowest link (Kramer and Evans 2011; Sharkey 2020). If we take the example of engineered plants reacting faster to changing light conditions, more energy from the light-dependent reaction is fed into the electron transport chain. However, if this additional energy cannot be processed by the electron transport chain (ETC), the rate of carbon fixation will be the same. In contrast, an overreduction of the ETC can create reactive oxygen species that will damage the photosynthetic apparatus and the cell, decreasing the overall photosynthetic productivity (Sonoike 2011; Vass 2012). This can at least in part explain the inconsistency in the results across species when they are engineered toward fluctuating light conditions. In one species (*Nicotiana tabacum*) the ETC was able to support the additional energy input from the light-dependent reaction, whereas in a different species (*Arabidopsis thaliana*) the reaction within the ETC are limiting and the extra light energy provided cannot be processed.

Thus, the ETC plays an important role in translating engineered photophysical and biochemical reactions of photosynthesis into improved biomass production in the field. To effectively engineer photosynthesis and maximize productivity, we need well-developed models to make informed decisions about what part of the ETC must be optimized to facilitate improved photosynthetic productivity and deliver consistent outcomes. In a recent article published in *Plant Physiology*, Gu (2023) derived photochemical equations based on kinetics of the redox reaction of the ETC, quantifying the linear electron transport capacity from PSII to PSI. Using these theoretical equations and combining them with measurements from diverse C3/C4 species across environments, Gu et al. identified several strategies that can increase the electron transport capacity of the ETC and contribute to faster growth of the photosynthetic organism.

**Figure 1.**
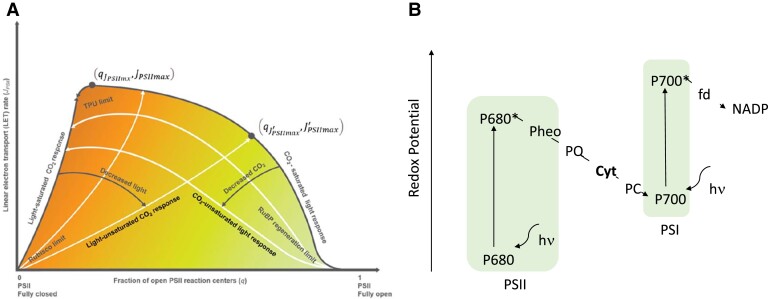
**A)** The 2-dimensional state space formed by the fraction of open PSII reaction centers (q) and the *J_PSII_*. The intersection between the CO_2_ response curve and the light response curve is the maximal rate of electron transport *J_PSIImax_*. Carboxylation limited by RuBP regeneration, Rubisco, and triose phosphate utilization (TPU) occupy the 3 corners of the state space. In general, bioengineered photochemical optimization can improve the efficiency of the ETC by shifting the q—JPSII state space up. A right shift is also desirable because it lowers the risk of creating reactive oxygen species. Adopted from Gu (2023). **B)** Simplified Z scheme of photosynthetic electron flow. After light absorption (hν) at P680 of PSII, the redox potential of the excited PS680* is elevated. An electron is then transferred through the electron transport chain to the Cyt. Light absorption of P700 and yields excited P700* of PSI, and an electron is passed onto ferredoxin (fd), a mobile iron sulfur protein and ultimately transferred onto NADP+ forming NADPH. The overall process creates a proton gradient across thylakoid membrane that is used by the ATP synthase for the production of ATP from ADP and inorganic phosphate.

The author first derived equations for a photochemical model of photosynthetic electron transport from a previous publication (Gu et al. 2023). This model has been tested against a dataset that consists of fluorometric measurements and gas exchange in response to changes in light, CO_2_, O_2_, and temperature of a variety of C3 and C4 species in Canada, China, the Netherlands, and USA (Han et al. 2022; Gu et al. 2023). These equations define a 2-dimensional state space of the electron transport capacity (*J*_PSII_) (Fig. 1A). This can be envisioned when examining the boarder defining extremes of this space. When all parameters are optimal, such as temperature and CO_2_ concentration, the electron transport within the ETC (*J*_PSII_) gradually rises with increasing light intensity. The curve starts at fully open (idle) PSII (1 on the x axis of Fig. 1A) and moves toward fully closed PSII (PSII that is not able to process additional energy; 0 on the x axis of Fig. 1A). This curve reaches maximum when it intersects with the light-saturated CO_2_ response curve. This starts out at fully closed PSII (0). This is because if there is no CO_2_ to be utilized for photosynthesis, photosystems cannot operate nor can any electron transport occur because there is nothing to pass the electrons onto. With rising CO_2_ concentration, PSII can pass on the light energy into the electron transport chain powering the biochemical reactions of CO_2_ fixation. Thus, some PSII will be open, able to accept and transform the energy of the light into chemical energy (we move from 0 toward 1 on the x axis). At the same time the *J*_PSII_ rises until the maximum is reached when the CO_2_ concentration is saturated.

Usually, JPSII will fall anywhere within the space under the curve of the maximal electron transfer because some parameters are not optimal, for example, ambient CO2 concentration or suboptimal temperature, which slows down photochemical reactions. Bioengineering should aim to increase JPSII to push it closer to the maximum. However, it should be noted that when a large fraction of PSII is closed, light energy can be passed onto oxygen, which can lead to the creation of reactive oxygen species and damage the photosynthetic apparatus. Thus, higher productivity can also be achieved by shifting JPSII away from a high percentage of closed PSII, as this will be healthier for the organism.

On this basis, Gu identified several targets for bioengineering to increase photosynthetic productivity. The cytochrome b6f complex (Cyt), for example, would be a great target (Fig. 1B). An increase of Cyt can increase the electron transfer rate because it increases the capacity of electrons that can be processed within the ETC. However, one concern with this approach could be that space within the thylakoid membrane might be limiting and thus Cyt cannot be easily increased without sacrificing a different photosynthetic component, which will likely have a negative impact on JPSII. Therefore, it might be worth including a parameter into the model that reflects the space/volume available within the thylakoids to better predict consequences of overexpressing individual components of the photosynthetic apparatus. Another finding in this study is that increasing the electron carrier plastoquinone (PQ) (Fig. 1B) might have positive effects on the rate of photosynthesis. The author noted that PQ is not only present in the thylakoid membrane but also in chloroplast envelope and plastoglobuli. Thus, the author concluded that efforts to increase PQ abundance should take into consideration its multifunctionality and multilocation characteristics. The model also suggests that bioengineering of redox reaction kinetics along the ETC in conjunction with that of Rubisco could be very valuable. Even though this might be a very difficult task as it potentially involves complete reengineering of these components, substantial increases in productivity can be achieved this way.

A significant fraction of PSII reaction centers could be QB-nonreducing (Vredenberg 2011), meaning they are unable to pass electrons from QA onto QB and thus cannot feed them into the ETC. There is currently little knowledge of why these reaction centers exist. Considering that there is a wide range of relative abundance of this PSII between photosynthetic organisms, photosynthetic productivity can be increased by changing the PSII in crop plants to a PSII with lower QB-nonreducing abundance.

In conclusion, with the help of the model, several targets could be identified that are worth engineering in order to increase photosynthetic productivity. Because the entanglement of photosynthetic parameters, it is difficult even with the current model to precisely predict the outcome when adjustments to the photosynthetic components are made. However, with more data, the model can be fine-tuned, making this a very valuable tool guiding future bioengineering efforts to increase photosynthetic productivity and thereby crop yields.
